# Supplementary material for: Associations of intimate partner violence and financial adversity with familial homelessness in pregnant and postpartum women: A 7-year prospective study of the ALSPAC cohort
Source: PLoS One. 2021 Jan 15;16(1):e0245507. doi: 10.1371/journal.pone.0245507 (PMC7810303; doi:10.1371/journal.pone.0245507)
Supplement: S1 Appendix — (DOCX) [file pone.0245507.s001.docx]

**S1 APPENDIX**

***S1 Table. Reporting periods of time-varying variables***

| **Wave** | **Age of child at time of reporting** | **Reporting period** | **Responses refer to incidents in the prior** |
| --- | --- | --- | --- |
| 1 | 18 weeks gestation | Since pregnancy | 18 weeks (4.14 months) |
| 2 | 8 weeks | Since mid-pregnancy | 27 weeks (6.21 months) |
| 3 | 8 months | Since child born | 8 months |
| 4 | 1 year 9 months | Since 8 months of age | 13 months |
| 5 | 2 years 8 months | Since 18 months of age | 15 months |
| 6 | 3 years 11 months | Since 30 months of age | 17 months |
| 7 | 5 years 1 month | Past year | 12 months |
| 8 | 6 years 1 month | Since child’s 5^th^ birthday | 13 months |

1. ***List of supplemental predictor variables used in MICE using FCS***

All additional predictors used in MICE using FCS were measured at baseline or as noted. Variables were imputed in order from least to most missing. The additional predictors were: partner’s highest level of education, partner’s social class, neighborhood quality index measured at wave 2, mother’s health in the last two weeks at 32 weeks gestation, life events score at 32 weeks gestation, child’s health in past month at 18 months of age, the Crown-Crisp Experiential Index (CCEI) total score and depression score at wave 3, whether the mother had financial difficulties at wave 3, household size at wave 3, mother’s alcohol consumption at wave 6, child’s total behavioral difficulties score at wave 6, number of children in the household at child’s age 7 years and one month, difficulty affording medical or dental care at wave 8, and child’s weight and height at age 8 years 9 months.

1. ***List of categorical predictor variables used in MICE using RF***

All continous predictors were categorized using the following cut-off values: Depression was defined as a CCEI depression score greater than or equal to 8, using a 15% prevalence estimate[40]; parity was grouped as zero, one, two, and three or more; age was categorized as below thirty, 30-34, 35-39, and forty or over[41]; child’s BMI was classed separately for boys and girls based on the CDC categories for underweight (less than 5^th^ percentile), normal or healthy weight (5^th^ to less than 85^th^ percentile), overweight (85^th^ to less than 95^th^ percentile), and obese (95^th^ percentile or greater); total children were classed as one, two, three, or four or more; household size was classed as two, three, four, or five or more; life events score was classed as zero, one, two, or three or more; child’s health score was classed as one, two, or three or more; neighborhood score was classed as less than 5, 5-7, 8-9, or ten or above; financial difficulties were categorized as none, 1-2, 3-7, or 8 or more; alcohol consumption was classed based on NIH standards for none, moderate, or heavy; postpartum depression was defined as an EPDS score of 13 or more[42]; CCEI score was grouped in quantiles 0-3, 4-6, 7-9, 10-14, and 15 or higher; low birthweight was defined as less than 2500 grams.

***S2 Fig. Prevalence of IPV, financial adversity, and homelessness***

| **A. Primary data - MICE using FCS**  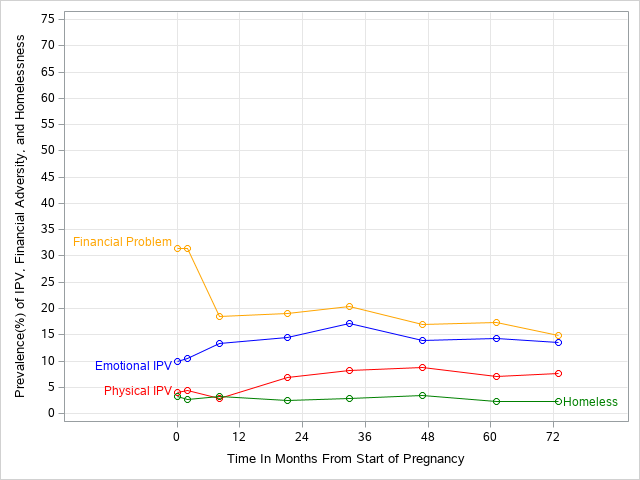 | |
| --- | --- |
| **B. Original Data**  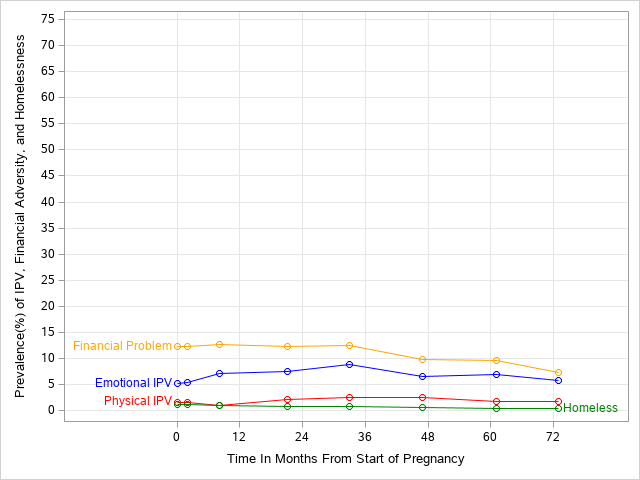 | **C. Secondary data - MICE using RF**  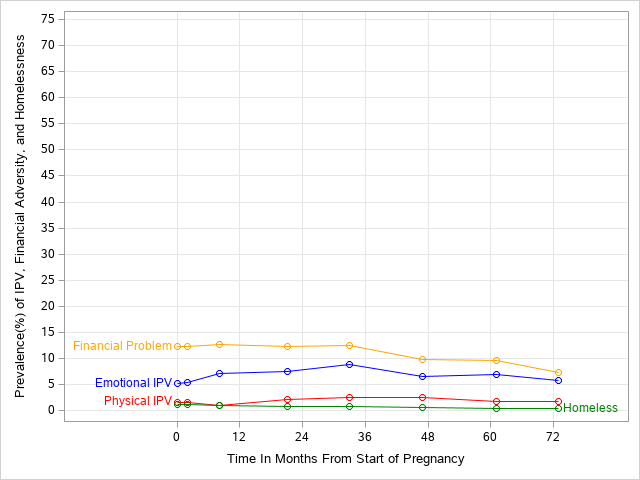 |

***S3 Fig. Prevalence of IPV, financial adversity, and homelessness, stratified by homeless status***

| **A. Primary data - MICE using FCS**  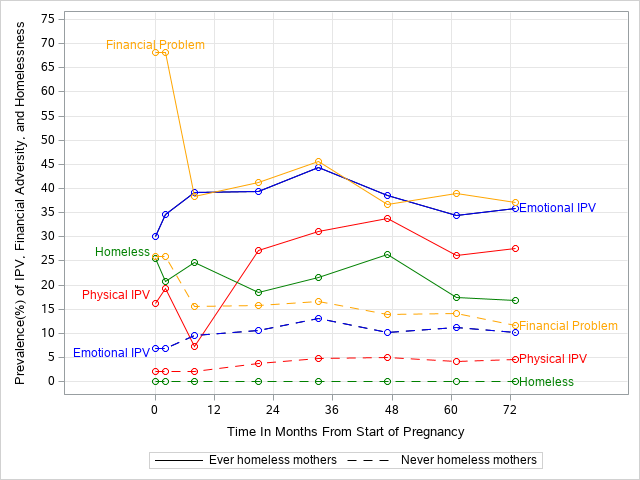 | |
| --- | --- |
| **B. Original Data**  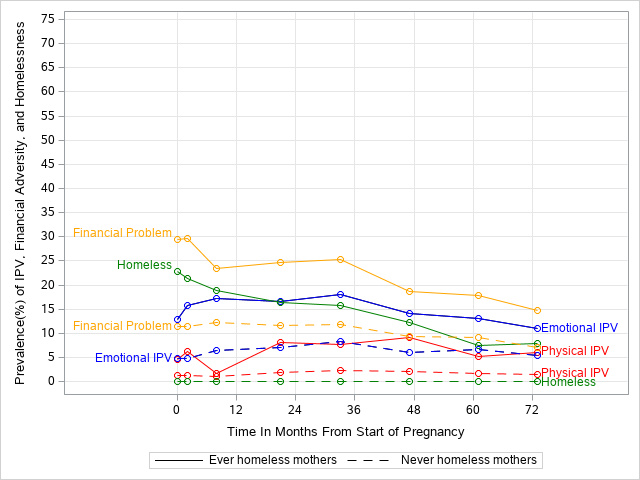 | **C. Secondary data - MICE using RF**  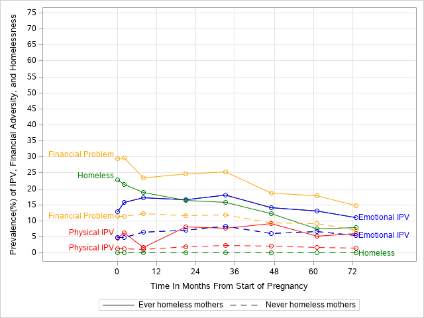 |

***S2 Table: Characteristics of study population in secondary data (MICE using RF)***

| **N = 14,734** | **Prevalence (%)** |
| --- | --- |
| ***Primary study variables*** |  |
| Any Physical IPV | 10.4% |
| Physical IPV: Wave 1 | 1.5% |
| Physical IPV: Wave 8 | 1.8% |
| Any Emotional IPV | 27.7% |
| Emotional IPV: Wave 1 | 5.3% |
| Emotional IPV: Wave 8 | 5.8% |
| Any major financial problem | 41.1% |
| Major financial problem: Wave 1 | 12.3% |
| Major financial problem: Wave 8 | 7.4% |
| Any homelessness | 5.2% |
| Homeless: Wave 1 | 1.2% |
| Homeless: Wave 8 | 0.4% |
| ***Socio-demographic variables*** |  |
| Age at delivery  Below 30  30-34  35-39  40 and over | 63.7%  26.7%  8.4%  1.2% |
| Ethnic background  White  Non-white | 97.7%  2.4% |
| Highest education qualification  CSE or less  Vocational  O level  A level  Degree | 14.5%  10.0%  39.8%  23.4%  12.4% |
| Social Class  I Professionals  II Managerial and technical  IIIN Skilled Non-manual  IIIM Skilled Manual  IV Partly skilled manual  V Unskilled Manual or Armed Forces | 4.9%  30.6%  46.8%  7.0%  8.8%  1.9% |
| Parity  Zero  One  Two  Three or more | 46.2%  34.9%  13.5%  5.5% |
| Edinburgh Post-Natal Depression Score  No  Yes | 92.7%  7.3% |
| Partner’s baseline alcoholism  No  Yes | 99.1%  0.9% |

***S4 Fig. Prevalence of IPV among mothers in 82 months following start of pregnancy, stratified by homeless status in RF imputed data***


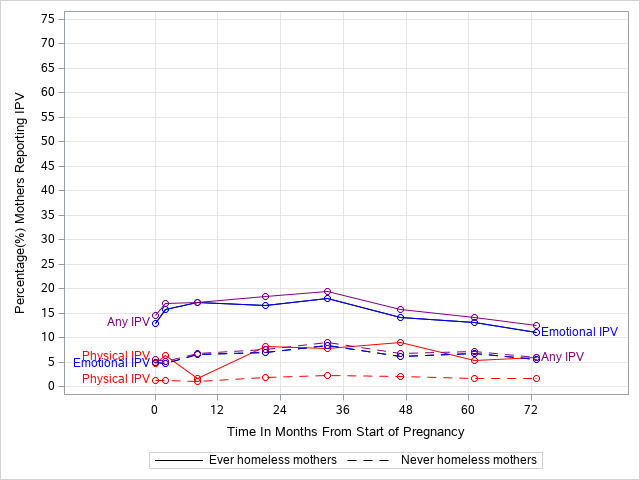


1. ***Results of Model 2 in FCS imputed data***

**Model 2**

Model 2 assumed that IPV occurs before IPV in each time interval. In Model 2, the estimated marginal effect of emotional IPV on incident homelessness over the study period was a 1.45 (95% CI: 1.17-1.81) times multiplicative increase in the discrete-time hazard of homelessness for every additional experience of emotional IPV. The estimated effect of each report of physical IPV was a 2.34 (95% CI: 1.47-3.72) times increase in homelessness. The estimated effect of each report of financial adversity was a 1.61 (95% CI: 1.46-1.77) times increase in homelessness.

The estimated marginal joint effect of emotional IPV and physical IPV on incident homelessness over the study period was a 2.81 (95% CI: 1.23-6.43) times multiplicative increase in the discrete-time hazard of homelessness for every additional experience of concurrent emotional IPV and financial adversity. The estimated effect of each report of concurrent emotional IPV and financial adversity was a 2.13 (95% CI: 1.45-3.14) times increase in homelessness. The estimated effect of each report of concurrent physical IPV and financial adversity was a 3.18 (95% CI: 1.53-6.63) times increase in homelessness.

The estimated marginal joint effect of emotional IPV, physical IPV, and financial adversity on incident homelessness over the study period was a 3.63 (95% CI: 1.10-12.02) times multiplicative increase in the discrete-time hazard of homelessness for every additional experience of concurrent emotional IPV, physical IPV, and financial adversity.

1. ***Results of sensitivity analysis on RF imputed data***

**Sample characteristics**

The imputed values from MICE using RF differed from the original data, but diverged unexpectedly from the values of the primary imputed data. The RF method consistently predicted that women with missing data were most similar to the majority or plurality category of each variable. The RF method predicted lower prevalence of physical and emotional IPV, financial adversity, and homelessness at each time point; however, it predicted higher prevalence of ever reporting these experiences across the entire study period. That is, more women experienced fewer incidents overall than in the original data. The RF imputed data characteristics were much closer to the original data than the FCS imputed data.

S4 Fig in the Appendix contrasts the life course trends of women who were homeless during the study period and those who were not, in the RF imputed data. The reported rates of exposure for homeless women are notably higher than for women who did not become homeless. Prevalence for both groups peaks at wave 5 (about 19% of ever homeless mothers, versus 8% of never homeless mothers). The most notable trend, unique to the group of ever homeless mothers, is a decrease in reported physical IPV in wave 3 (at a prevalence of 2%). This decrease is not mirrored in the reported rates of emotional IPV.

**Model 1 with RF imputed data**

Within the RF imputed data, Model 1 estimated that the marginal effect of emotional IPV on incident homelessness over the study period was a 1.77 (95% CI: 1.51-2.08) times multiplicative increase in the discrete-time hazard of homelessness for every additional experience of emotional IPV. The estimated marginal effect of each report of physical IPV on homelessness over the study period was a 4.12 (95% CI: 2.98-5.68) times increase in homelessness. The estimated marginal effect of each report of financial adversity was a 1.81 (95% CI: 1.67-1.95) times increase in homelessness.

The estimated joint effect of emotional and physical IPV on incident homelessness over the study period was a 4.84 (95% CI: 2.49-9.42) times multiplicative increase in the discrete-time hazard of homelessness for every additional experience of concurrent emotional and physical IPV. The estimated effect of each report of concurrent emotional IPV and financial adversity was a 2.67 (95% CI: 1.90-2.75) times increase in homelessness. The estimated effect of each report of concurrent physical IPV and financial adversity was a 5.55 (95% CI: 2.93-10.53) times increase in homelessness.

The estimated joint effect of emotional IPV, physical IPV, and financial adversity on incident homelessness over the study period was a 5.92 (95% CI: 1.85-18.97) times multiplicative increase in the discrete-time hazard of homelessness for every additional experience of concurrent emotional IPV, physical IPV, and financial adversity.

**Model 2 with RF imputed data**

Within the RF imputed data, Model 1 estimated that the marginal effect of emotional IPV on incident homelessness over the study period was a 1.77 (95% CI: 1.51-2.07) times multiplicative increase in the discrete-time hazard of homelessness for every additional experience of emotional IPV. The estimated marginal effect of each report of physical IPV was a 4.25 (95% CI: 3.05-5.91) times increase in homelessness. The estimated marginal effect of each report of financial adversity was a 1.80 (95% CI: 1.66-1.94) times increase in homelessness.

The estimated joint effect of emotional IPV and physical IPV on incident homelessness over the study period was a 5.05 (95% CI: 2.62-9.72) times multiplicative increase in the discrete-time hazard of homelessness for every additional experience of concurrent emotional IPV and financial adversity. The estimated effect of each report of concurrent emotional IPV and financial adversity was a 2.69 (95% CI: 1.94-3.73) times increase in homelessness. The estimated effect of each report of concurrent physical IPV and financial adversity was a 6.25 (95% CI: 3.25-12.03) times increase in homelessness.

The estimated joint effect of emotional IPV, physical IPV, and financial adversity on incident homelessness over the study period was a 6.66 (95% CI: 2.12-20.91) times multiplicative increase in the discrete-time hazard of homelessness for every additional experience of concurrent emotional IPV, physical IPV, and financial adversity.
