# Supplementary material for: Associations of intimate partner violence and financial adversity with familial homelessness in pregnant and postpartum women: A 7-year prospective study of the ALSPAC cohort
Source: PLoS One. 2021 Jan 15;16(1):e0245507. doi: 10.1371/journal.pone.0245507 (PMC7810303; doi:10.1371/journal.pone.0245507)
Supplement: Attachment — Submitted filename: Response to Reviewers.docx [file pone.0245507.s002.docx]

December 8, 2020

Marianna Mazza
Academic Editor
PLOS ONE

Dear Dr. Marianna Mazza,

We would like to thank you for your response letter and the opportunity to resubmit a revised copy of this manuscript. We express our thanks for the positive feedback and helpful comments for correction and modification. We believe the modifications have resulted in an improved revised manuscript, which you will find uploaded alongside this document. The manuscript has been revised to address the reviewer comments. We state below the steps we have taken to address the points raised in your letter.

We very much hope the revised manuscript is accepted for publication in PLOS ONE.

Sincerely,

Caitlin Chan, on behalf of the authors

Journal Requirements:

When submitting your revision, we need you to address these additional requirements.

1. Please ensure that your manuscript meets PLOS ONE's style requirements, including those for file naming. The PLOS ONE style templates can be found at

https://journals.plos.org/plosone/s/file?id=wjVg/PLOSOne_formatting_sample_main_body.pdf and

https://journals.plos.org/plosone/s/file?id=ba62/PLOSOne_formatting_sample_title_authors_affiliations.pdf

 We corrected the formatting on the title page. We modified the spacing in the tables, the formatting of the table and figure citations, and the order of the paper sections.

2. Thank you for including your ethics statement:

"Ethical approval for the study was obtained from the ALSPAC Ethics and Law Committee and the Local Research Ethics Committees (Reference number C2481), and all participants provided written informed consent."

a. Please amend your current ethics statement to include the full name of the ethics committee/institutional review board(s) that approved your specific study.
b. Once you have amended this/these statement(s) in the Methods section of the manuscript, please add the same text to the “Ethics Statement” field of the submission form (via “Edit Submission”).

For additional information about PLOS ONE ethical requirements for human subjects research, please refer to <http://journals.plos.org/plosone/s/submission-guidelines#loc-human-subjects-research>."

We have revised the ethics statement as follows:

“Ethical approval for the study was obtained from the ALSPAC Ethics and Law Committee and the Local Research Ethics Committees (Reference number C2481), and all participants provided written informed consent. ALSPAC Executive committee has been registered with/has an Institutional Review Board status with the United States Department of Health and Human Services and Office for Human Research Protections (IRB I00003312). The ALSPAC committee approval serves as the IRB approval. The full list of Research Ethics Committees who approved the study can be accessed online (http://www.bristol.ac.uk/media-library/sites/alspac/documents/governance/Research%20Ethics%20Committee%20approval%20references.pdf)”

This statement has been updated in both the methods section and the ethics statement of the submission file.

3. In your Methods section, please provide additional information about the participant recruitment method and the demographic details of your participants. Please ensure you have provided sufficient details to replicate the analyses such as:

a) the recruitment date range (month and year),

b) a description of how participants were recruited, and c) descriptions of where participants were recruited and where the research took place.

 We added the recruitment location, date range, and additional description of the recruitment process of the original cohort. We further moved the study exclusion criteria from the appendix to the methods section of the paper.

4. Please include additional information regarding the survey or questionnaire used in the study and ensure that you have provided sufficient details that others could replicate the analyses. For instance, if you developed a questionnaire as part of this study and it is not under a copyright more restrictive than CC-BY, please include a copy, in both the original language and English, as Supporting Information.

 We have included in the methods section a link to the questionnaires.

5. We note that you have indicated that data from this study are available upon request. PLOS only allows data to be available upon request if there are legal or ethical restrictions on sharing data publicly. For information on unacceptable data access restrictions, please see http://journals.plos.org/plosone/s/data-availability#loc-unacceptable-data-access-restrictions.

In your revised cover letter, please address the following prompts:

a) If there are ethical or legal restrictions on sharing a de-identified data set, please explain them in detail (e.g., data contain potentially identifying or sensitive patient information) and who has imposed them (e.g., an ethics committee). Please also provide contact information for a data access committee, ethics committee, or other institutional body to which data requests may be sent.

b) If there are no restrictions, please upload the minimal anonymized data set necessary to replicate your study findings as either Supporting Information files or to a stable, public repository and provide us with the relevant URLs, DOIs, or accession numbers. Please see http://www.bmj.com/content/340/bmj.c181.long for guidelines on how to de-identify and prepare clinical data for publication. For a list of acceptable repositories, please see http://journals.plos.org/plosone/s/data-availability#loc-recommended-repositories.

We will update your Data Availability statement on your behalf to reflect the information you provide.

Access to ALSPAC research data must be requested using the formal procedures described in this document (http://www.bristol.ac.uk/media-library/sites/alspac/documents/researchers/data-access/ALSPAC_Access_Policy.pdf) and is subject to eligibility, the ALSPAC funder’s terms and conditions and University of Bristol policies and procedures. Data are available upon request from the ALSPAC executive committee (alspac-exec@bristol.ac.uk) for researchers who meet the criteria for access.

[Note: HTML markup is below. Please do not edit.]

Reviewers' comments:

Reviewer's Responses to Questions

**Comments to the Author**

1. Is the manuscript technically sound, and do the data support the conclusions?

The manuscript must describe a technically sound piece of scientific research with data that supports the conclusions. Experiments must have been conducted rigorously, with appropriate controls, replication, and sample sizes. The conclusions must be drawn appropriately based on the data presented.

Reviewer #1: Yes

2. Has the statistical analysis been performed appropriately and rigorously?

Reviewer #1: Yes

3. Have the authors made all data underlying the findings in their manuscript fully available?

The [PLOS Data policy](http://www.plosone.org/static/policies.action#sharing) requires authors to make all data underlying the findings described in their manuscript fully available without restriction, with rare exception (please refer to the Data Availability Statement in the manuscript PDF file). The data should be provided as part of the manuscript or its supporting information, or deposited to a public repository. For example, in addition to summary statistics, the data points behind means, medians and variance measures should be available. If there are restrictions on publicly sharing data—e.g. participant privacy or use of data from a third party—those must be specified.

Reviewer #1: Yes

4. Is the manuscript presented in an intelligible fashion and written in standard English?

PLOS ONE does not copyedit accepted manuscripts, so the language in submitted articles must be clear, correct, and unambiguous. Any typographical or grammatical errors should be corrected at revision, so please note any specific errors here.

Reviewer #1: Yes

5. Review Comments to the Author

Please use the space provided to explain your answers to the questions above. You may also include additional comments for the author, including concerns about dual publication, research ethics, or publication ethics. (Please upload your review as an attachment if it exceeds 20,000 characters)

Reviewer #1: The manuscript describes the relationship of intimate partner violence and financial adversity with familial homelessness in pregnant and postpartum women.
The theme is adequately explored and with a flowing form. It could be stimulating a reflection on the victimization process and the connected psychopathological configurations, as well as on the fact that challenging circumstances and resilience skills can contribute to a better coping with stress and adversity.
It might be interesting to analyze the impact of COVID19 pandemic on emotional distress and sexuality. It would therefore be useful to read and discuss the following papers:

G Marano, E Gaetani, A Gasbarrini, L Janiri, G Sani, M Mazza, Multidisciplinary Gemelli Group for HHT. Mental health and counseling intervention for hereditary hemorrhagic telangiectasia (HHT) during the COVID-19 pandemic: perspectives from Italy. Eur Rev Med Pharmacol Sci. 2020 Oct;24(19):10225-10227. doi: 10.26355/eurrev_202010_23246.

M Mazza, G Marano, L Janiri, G Sani. Managing Bipolar Disorder patients during COVID-19 outbreak. Bipolar Disord. 2020 Oct 4. doi: 10.1111/bdi.13015.

M Mazza, G Marano, B Antonazzo, E Cavarretta, M Di Nicola, L Janiri, G Sani, G Frati, E Romagnoli. What about heart and mind in the covid-19 era? Minerva Cardioangiol. 2020 May 12. doi: 10.23736/S0026-4725.20.05309-8.

M Mazza, G Marano, C Lai, L Janiri, G Sani. Danger in danger: Interpersonal violence during COVID-19 quarantine. Psychiatry Res. 2020 Jul;289:113046. doi: 10.1016/j.psychres.2020.113046.

We cited the last paper, “Danger in danger: Interpersonal violence during COVID-19 quarantine,” which discusses a potential rise in interpersonal violence that may exacerbate the conditions discussed in our study. The other studies are not directly relevant to the subject matter of our paper.

6. PLOS authors have the option to publish the peer review history of their article ([what does this mean?](https://journals.plos.org/plosone/s/editorial-and-peer-review-process#loc-peer-review-history)). If published, this will include your full peer review and any attached files.


If you choose “no”, your identity will remain anonymous but your review may still be made public.


**Do you want your identity to be public for this peer review?** For information about this choice, including consent withdrawal, please see our [Privacy Policy](https://www.plos.org/privacy-policy).

Reviewer #1: No

[NOTE: If reviewer comments were submitted as an attachment file, they will be attached to this email and accessible via the submission site. Please log into your account, locate the manuscript record, and check for the action link "View Attachments". If this link does not appear, there are no attachment files.]

While revising your submission, please upload your figure files to the Preflight Analysis and Conversion Engine (PACE) digital diagnostic tool, <https://pacev2.apexcovantage.com/>. PACE helps ensure that figures meet PLOS requirements. To use PACE, you must first register as a user. Registration is free. Then, login and navigate to the UPLOAD tab, where you will find detailed instructions on how to use the tool. If you encounter any issues or have any questions when using PACE, please email PLOS at [figures@plos.org](mailto:figures@plos.org). Please note that Supporting Information files do not need this step.
